# Comparison of Intradialytic Parenteral Nutrition with Glucose or Amino Acid Mixtures in Maintenance Hemodialysis Patients

**DOI:** 10.3390/nu8060220

**Published:** 2016-06-02

**Authors:** Yan Liu, Xiao Xiao, Dan-Ping Qin, Rong-Shao Tan, Xiao-Shi Zhong, Dao-Yuan Zhou, Yun Liu, Xuan Xiong, Yuan-Yuan Zheng

**Affiliations:** 1 Department of Nephrology, Guangzhou Red Cross Hospital, Fourth Affiliated Hospital of Medical College of Jinan University, No. 396 Tong Fu Zhong Road, Guangzhou 510220, China; XiaoXiao9943@sina.com (X.X.); DanpingQin11@163.com (D.-P.Q.); XiaoshiZhong33@163.com (X.-S.Z.); daoyuanzhou15@sina.com (D.-Y.Z.); yunliu0223@sina.com (Y.L.); xuanxiong10@sina.com (X.X.); yuanyuanzheng22@sina.com (Y.-Y.Z.); 2 Guangzhou Institute of Disease-Oriented Nutritional Research, Guangzhou 510220, China; rongshaotan01@sina.com

**Keywords:** chronic kidney disease, glucose supplement, hemodialysis, IDPN, nutritional intervention, amino acid supplementation, hemodialysis, protein energy wasting, PEW

## Abstract

Many long-term maintenance hemodialysis patients have symptoms of protein-energy wasting caused by malnutrition. Each session of hemodialysis removes about 10 to 12 g of amino acids and 200 to 480 kcal of energy. Patients receiving hemodialysis for chronic kidney disease may be undernourished for energy, protein consumption, or both. Non-diabetic hemodialysis patients were randomized to three treatment groups: oral supplementation, oral supplementation plus high-concentration glucose solution (250 mL containing 50% glucose) and these two interventions plus 8.5% amino acids solution. The post-treatment energy status of the glucose group was significantly higher than its baseline level, whereas the control group’s status was significantly lower. The glucose group had significantly higher concentrations of asparagine, glutamine, glycine, alanine, and lysine after treatment. All treatment groups had significantly increased hemoglobin levels but significantly decreased transferrin levels after treatment compared to baseline. After treatment, the amino acid group had significantly higher albumin level compared to the glucose group (*p* = 0.001) and significantly higher prealbumin level compared to the control group (*p* = 0.017). In conclusion, long-term intervention with high-concentration glucose solution at each hemodialysis session is a simple and cheap method that replenished energy stores lost during hemodialysis of non-diabetic patients.

## 1. Introduction

Fatigue and muscle wasting are two common challenges of chronic dialysis patients. Muscle wasting affects about 18% to 75% of patients with chronic kidney disease who are undergoing maintenance dialysis [1,2]. Protein-energy wasting (PEW) is diagnosed if a patient has low serum levels of cholesterol, albumin, or transthyretin; reduced body mass; and less muscle mass observed by mid-arm muscle circumference [1,3] Causes of PEW include uremia, dialysis intervention (nutrient loss and biocompatibility), inadequate nutrient (protein and energy) consumption, hypermetabolism (micro-inflammation), hormone dysregulation (hyperparathyroidism, insulin-resistance, and insulin growth factor 1 resistance), and social and psychological factors [3,4]. Lower protein and/or energy intakes than recommended for patients on hemodialysis are associated with higher morbidity and mortality [5]. Hemodialysis or peritoneal dialysis of these patients can exacerbate inadequate consumption of nutrients. Each session of hemodialysis reduces the free amino acid levels in plasma by 30%, losing about 10 to 12 g of amino acids [4,6]. Hemodialysis differentially causes the loss of branched-chain amino acids to a greater extent than aromatic amino acids [7]. Hemodialysis also removes approximately 200–480 kcal each session [4,6].

Patients receiving hemodialysis may have good nutritional status [7] or may have become undernourished for energy intake, undernourished for protein consumption, leading to catabolism of muscle tissue and muscle wasting, or both [8]. Patients with PEW often receive nutritional counseling and education, oral nutritional supplements, and nutritional intervention by parenteral or enteral routes. Benefits are observed in patients with chronic kidney disease and malnutrition. Higher protein diets (1.4 g/kg/day) increased or maintained protein synthesis on days without dialysis whereas low protein diets (0.5 g/kg/day) did not. The highest survival rates were associated with normalized protein intake of 1 to 1.4 g/kg/day [9], which may activate protein metabolism and change BUN (blood urea nitrogen) metabolic dynamics prior to hemodialysis. Patients with normalized protein intakes of less than 0.8 g/kg/day or those with protein intakes greater than 1.4 g/kg/day showed higher mortality rates [9]. Administration of the intradialytic parenteral nutrition (IDPN), a “three-in-one” solution containing glucose/dextrose, amino acids, and fat emulsion, appears to help malnourished patients with CKD by increasing protein (albumin) synthesis [10,11]. IDPN usually is administered for six months or less and approximately 23% reached the stated nutritional goals for switching to solely oral supplementation [11]. Some patients discontinued due to excess fluid gain (23%), nausea (4%), uncontrollable hyperglycemia (4%), kidney transplant (23%), death (23%), transfer, or other causes [11]. Some substances in the mixture may not be needed to support specific patients.

Fluid overload is defined as excess extracellular water/total body water (≥0.40) and is an important predictor of mortality in continuous ambulatory peritoneal dialysis [12].

A simple, effective, safe, and economic method to treat malnutrition in non-diabetic hemodialysis patients is needed. This prospective controlled study aimed to investigate the safety and effectiveness of glucose injection during dialysis for the treatment of malnutrition of dialysis patients. The current study compared three interventions in non-diabetic patients: oral supplementation (control), oral supplementation plus high-concentration glucose solution (250 mL containing 50% glucose; glucose group), and these two interventions plus amino acids solution (amino acid group). The parameters currently evaluated included SAG, blood biochemistry, and blood amino acid profile. We postulated that supplemental glucose would improve amino acid metabolism, increase nitrogen reuse, and improve overall nutritional status. We currently report that long-term intervention of high-concentration glucose solution (250 mL containing 50% glucose) at each hemodialysis session provides a simple and inexpensive method to replenish energy stores lost by hemodialysis while avoiding side effects such as an increased hydration load due to infusion of a liter of fluid.

## 2. Materials and Methods

### 2.1. Patients

This prospective, 2 armed, controlled study interviewed 113 hemodialysis patients, of which 36 non-diabetic patients were enrolled in the study.

The study was conducted between July 2012 and May 2013. Inclusion criteria were as follows: patients received dialysis in our center for more than 3 months; the mean duration of dialysis was 3.7 ± 0.98 years (control group: 3.6 ± 1.1 years; glucose groups: 3.9 ± 0.49 years and 3.7 ± 0.56 years). There were no marked differences among three groups. Subjects also needed to be adults (age > 18 years), continuous hemodialysis for more than three months without parenteral nutrition intervention or oral enteral nutrition intervention; and a body mass index (BMI) >18. Additional inclusion criteria were one or more biochemical markers with the values: serum albumin (ALB) <35 g/L; serum prealbumin (PA) <200 mg/L; serum transferrin (TRF) <200 mg/dL; or normalized protein decomposition/protein catabolic rate (PCR) <1.1 g/kg·d. Patients with hypertension, anemia, calcium or phosphate metabolic disorders were allowed. Exclusion criteria were diabetes, infectious diseases (e.g., hepatitis and tuberculosis), cancer, other wasting diseases, heart failure, gastrointestinal diseases; co-morbidities besides hypertension, anemia, calcium or phosphate metabolic disorders; immediate medical need for surgery or surgery within 3 months; and no informed consent. The study protocol (IRB) was approved by the committee of Guangzhou Red Cross Hospital of Guangdong Province (approval No. 20120614) and patients provided written informed consent.

Nutritional Supplements: The solutions containing nutritional supplements, 8.5% amino acids (Novamin; 0.63 g aspartic acid; 1.05 g each of serine, glutamate, threonine, methionine, and isoleucine; 1.48 g each of glycine, phenylalanine, and leucine; 1.25 g each of histidine and proline; 3.05 g alanine; 0.08 g tyrosine; 1.38 g valine; 2.38 g lysine; 0.35 g tryptophan; amino acids 21.25 g; nitrogen 3.5 g; total energy 87.5 Kcal) and 50% glucose (size: 250 mL), were purchased from Sino-Swed Pharmaceutical Corp, LTD (Wuxi City, Jiangsu Province, China). The dialysate used the Fresenius’ dialysis prescription: glucose-free bicarbonate dialysate.

### 2.2. Energy Intake and Fluid Overload

Patients maintained a food record for three continuous days before treatment and after treatment. The energy intake for each food was calculated, and the mean energy intake per day pretreatment and post-treatment (±standard deviation (SD)) were calculated. Group energy intake was expressed as median and inter-quartile range. Fluid overload was monitored by comparison of the dry weight of patients.

### 2.3. The Treatment Protocol

The 36 non-diabetic patients were assigned to three groups based on a table of random numbers and the interventions were performed alongside hemodialysis for 9 months. Each group included 12 patients. Control group received routine nutrition education, oral nutritional intervention, adequate dialysis, erythropoietin (10,000 IU) weekly, and correction of acidosis. In addition, nutritional intervention was adjusted for each individual and included: (i) activated vitamin D adjusted for serum parathyroid hormone concentrations and calcium serum concentration; (ii) oral or intravenous iron supplement adjusted for state of anemia; and (iii) water-soluble vitamin mixture. Second, the 50% glucose intervention group received the treatments provided in the control group plus 250 mL of 50% glucose solution was given during each hemodialysis (equivalent to 2512.08 KJ(600.69Kcal), Sino-Swed Pharmaceutical Corp. Ltd.). Third, the 8.5% amino acid intervention group received all treatments plus 250 mL of 8.5% amino acid solution during each hemodialysis. Notably, the amino acid intervention group did not receive glucose solution. The parenteral nutrition intervention in all cases was started 0.5 h after the beginning of dialysis. The fluid was injected slowly (<1.5 mL/min) but continuously for 3 h via the arterial port using a blood pump, which was clamped after infusion to prevent air entering the blood circulation. Blood glucose levels in the fingertips were measured before, during, and after glucose treatment for diabetes patients who were receiving 50% glucose treatment. The duration of the study was 9 months.

### 2.4. Sample Collection and Analysis

The pre-dialysis blood samples were obtained before hemodialysis and the post-dialysis blood samples were collected after hemodialysis without access to the recirculation. Blood pump sampling technique was used to slow down or terminate the process. The blood samples were allowed to clot for 10 min., chilled at 4 °C, and centrifuged. Serum was harvested and stored at 4 °C. Pre and post dialysis samples were sent for biochemical examination to the Guangzhou Analytical Center within 6 h for detecting the amino acid spectrum. Biochemical parameters (TRF (transferrin), PA (prealbumin), TP (total protein), ALB (albumin), BUN (blood urea nitrogen), Cr (creatinine), TC (total cholesterol), TG (triglycerides), LDL-C (LDL-cholesterol), hypersensitive C- reactive protein (hsCRP) and Ca (calcium)) using a Hitachi 7600 automatic biochemical analyzer. Serum free amino acid spectrum was measured with pre-column *o*-phthalaldehyde-9-fluorenylmethyloxycarbonyl derivatization and reversed-phase high-performance liquid chromatography using the HP 1050 HPLC system. The blood glucose levels were monitored every hour during dialysis using finger-stick testing. The HBA1c levels were not monitored routinely because patients were not diabetic (diabetes was an exclusion criteria). Upper gastrointestinal and stool examinations were not performed routinely. Adequacy of hemodialysis, which measures the clearance of urea from the blood and peripheral fluid compartment (in tissues), was expressed by the ratio Kt/V (clearance of urea (K) multiplied by time (t) of dialysis provides the dialysis volume; V distribution volume of urea). Kt/V of 1.0 means that dialysis had cleared urea from a volume of blood equal to the distribution volume of urea. Kt/V was calculated as follows:

Kt/V = −Ln(R-0.008 × t) + (4-3.5 × R) × UF/W

where Ln is the natural log, R is post-dialysis blood urea nitrogen (BUN)/predialysis BUN, t is the length of dialysis, UF is ultrafiltration volume of dialysis, and weight is post-dialysis body weight.

### 2.5. Statistical Analysis

Age, weight, and BMI were presented as mean and standard deviation, the one-way ANOVA test was performed to compare the differences among the three treatment groups. Other continuous data were presented by median and inter-quartile range (IQR) due to the small sample size and non-normal distributions. The non-parametric Kruskal–Wallis test was performed to compare the three treatment groups, and the *post-hoc* tests between each set of two treatment groups was performed by the non-parametric Mann–Whitney test with Bonferroni correction. The difference between baseline and post-treatment within treatment groups were tested with the non-parametric Wilcoxon signed ranks test. Categorical data (SGA and gender) are presented by count and percentage and the Fisher’s exact test was performed to test the difference between treatment groups. The McNemar’s test was used to test the change of subjective global nutritional assessment (SGA) from baseline to post-treatment. A two-sided *p*-value less than 0.05 indicated statistical significance. The statistical analyses were assessed using the software IBM SPSS Statistics 19.0 (IBM Corporation, Armonk, NY, USA).

## 3. Results

### 3.1. Patients

Thirty-six of the 113 non-diabetic hemodialysis patients interviewed were placed randomly into one of the three treatment groups. Four patients were transferred to another hospital and did not complete the study. Thus, 32 patients (18 females and 14 males) were analyzed. Their renal insufficiency had been diagnosed as primary chronic glomerular disease (*n* = 13), hypertensive nephropathy (*n* = 12), obstructive nephropathy (*n* = 4), or interstitial nephritis (*n* = 3). Baseline characteristics (age, gender, weight, BMI, dialysis frequency and duration, comorbid conditions, SGA nutritional status, energy intake, biochemical markers and amino acid concentrations) were similar among the three groups, except for TCO_2_ and the concentration of proline (Table 1, Table 2 and Table 3). During the nine-month study period, each patient received appropriate treatment for every dialysis treatment (one dose), three treatments per week (three doses/week). The internal fistula was used for vascular access in 78% of patients, and a double-lumen catheter with cuff was used as a vascular access in the remaining 22% of patients. Blood flow ranges were found to be between 200 and 300 mL/min. There was no significant difference among three groups. Hospitalization and infection related to study therapies were not observed during the study period. The average duration of dialysis for all patients was 3.7 ± 0 98 years (control group: 3.6 ± 1.1, glucose group: 3.9 ± 0.49, and amino acid group 3.7 ± 0.56); no statistical difference among three groups.

### 3.2. Nutritional Status

At baseline, 19 patients had SGA (subjective global nutritional assessment) classification of A (very mild risk to well-nourished), 10 patients had SGA classification of B (mild to moderate malnourished), and three had SGA classification of C (severely malnourished). After treatment, there was no significant change of SGA nutritional status compared to baseline, in each of the three treatment groups. No significant difference was observed between the three treatment groups (Table 2).

### 3.3. Energy Intake

The difference of energy intake between baseline and post-treatment significantly differed among the control and glucose groups (Table 2). The post-treatment energy intake of the glucose group was significantly increased compared to its baseline level (medians from 23.9 to 28.2 kcal/kg, *p* = 0.037). In contrast, the post-treatment energy intake of the control group was significantly less than its baseline level (medians from 25.3 to 24.9 kcal/kg, *p* = 0.048) (Table 2). The energy intake reduced post treatment in the amino acid group may be due to gastrointestinal irritation caused by amino acid injection, which affects food/caloric intake. In amino acid group, supplementation with additional glucose was not performed. The body weight before a dialysis increased as compared to that after last dialysis (dry body weight). If it is not higher than 5% of dry body weight (or 2.5 kg) in a specific patient, overload is defined if higher than 5% of dry body weight (or 2.5 kg) is observed after consecutive three dialyses.

### 3.4. Biochemical Indices

Table 3 compared the changes in the median values for the biochemical indices as a function of time for the three groups. The parameters of renal function, BUN, Kt/V (a measure of efficiency of dialysis), creatinine, and serum creatinine were compared among groups. Patients in the glucose group had significantly increased BUN levels after treatment compared to baseline (*p* = 0.049). The glucose group had a significantly lower median Kt/V level after treatment compared to baseline (*p* = 0.035). In contrast, the amino acid group had a significantly higher median Kt/V level after treatment compared to baseline (*p* = 0.034). The change of Kt/V from baseline to post-treatment differed significantly among the three treatment groups (*p* = 0.007). Patients in the amino acid group had significantly increased serum creatinine and Calcium x phosphate levels after treatment compared to baseline (median SCr: *p* = 0.034; median Ca × p: *p* = 0.016).

The effect of nutritional intervention on protein synthesis was indicated in the hemoglobin (Hb), transferrin (TRF), and albumin values. All treatment groups had significantly increased Hb levels but significantly decreased TRF levels after treatment compared to baseline (*p* < 0.05). The changes in Hb and TRF from baseline to post-treatment did not differ among the three treatment groups (*p* > 0.05). After treatment, the amino acid group had significantly higher albumin level compared to the glucose group (*p* = 0.001).

None of the treatments affected the total cholesterol (TC) levels. The control and glucose groups had significantly decreased triglyceride (TG) levels after treatment (control group: *p* = 0.020; glucose group: *p* = 0.002). The control group had significantly decreased LDL-C and TCO_2_ levels after treatment (median LDL-C: *p* = 0.004; median TCO_2_: *p* = 0.018). The glucose and amino acid groups had significantly increased Ca^2+^ levels after treatment (glucose group: *p* = 0.018; amino acid group: *p* = 0.001) (Table 3). All patients in the three groups survived and glucose concentrations were stable.

### 3.5. Concentrations of Amino Acids

The median amino acid concentrations as a function of time and supplementation are listed in Table 4. After treatment, the control group had no significant change in the amino acid concentrations compared to baseline, except for a significant decrease in arginine (*p* = 0.049) (Table 4). The glucose group had significantly increased the concentrations of asparagine (*p* = 0.002), glutamine (*p* = 0.010), glycine (*p* = 0.020), alanine (*p* = 0.021), and lysine (*p* = 0.037) (Table 4).

In contrast, the amino acid group had significantly lower levels after treatment for concentrations of aspartic acid (*p* = 0.013), alanine (*p* = 0.013), methionine (*p* = 0.028), phenylalanine (*p* = 0.020), and proline (*p* = 0.041) (Table 4). Furthermore, the changes from baseline of asparagine, glycine, histidine, alanine, methionine, and phenylalanine were significantly different between the glucose and the amino acid groups (all *p* < 0.0167) (Table 4).

## 4. Discussion

In the present study, we provide a 50% glucose solution as energy support for non-diabetic hemodialysis patients who usually lose 200 kcal to 480 kcal energy during each dialysis. This supplement is not expensive and is demonstrated to be effective in non-diabetic patients, such that it provides an overall improved nutritional state. In addition, most biochemical indices such as the level of hsCRP, TG, and TC did not fluctuate significantly before and after treatment, indicating that the treatment described in this study is safe for non-diabetic hemodialysis patients at least for short periods of time. Many studies have investigated administration of IDPN that included glucose/dextrose, amino acids, and fat emulsion [13,14]. This type of nutrition is suitable for malnourished patients who cannot receive oral nutritional support. The total volume of these parenteral administered nutrients is relatively large, ranging from 750 mL to 1000 mL at every dialysis (3–4 times per week), although it adds only 2% to 4% water volume to the total body water volume at each session (males 32 L to 44 L; females 23.9 L to 33.2 L) [15].

Fluid overload (≥7%) in patients with chronic kidney disease is an independent risk factor for cardiovascular morbidity and mortality of any cause [16]. In our study, four patients (two in control and two in glucose groups) had transferred and discontinued treatment in this study; they had not shown any trend towards fluid overload. In comparison, a previous study of 26 malnourished hemodialysis patients in Vancouver, Canada [11] reported that six of 26 malnourished hemodialysis patients (23%) had to discontinue IDPN, which adds 1 L fluid every session, due to excess fluid buildup. Most of the Canadian malnourished hemodialysis patients (18/26, 69%) did not complete the full nine courses of IDPN due to excess fluid weight gain (*n* = 6), kidney transplant (*n* = 4), death (*n* = 4), uncontrolled hyperglycemia (*n* = 1), and nausea (*n* = 1) [11]. Moreover, the most efficient methods for improving the energy and nutritional status of hemodialysis patients is not yet determined. One advantage of the 50% glucose solution infusion to the non-diabetic hemodialysis patients is its lower volume, which may reduce the tendency for fluid overload.

Interestingly, patients in the glucose group had significantly decreased Kt/V level after treatment. Although the observation needs confirmation and the mechanism is not known, one possible explanation is that the complemented glucose promotes protein synthesis *in vivo*, which increases the endogenous urea nitrogen content (lowering Kt/V). Kt/V was calculated according to the Volume of urea nitrogen distributed in patients receiving dialysis. There is evidence showing that, when the caloric supply is enough, the positive nitrogen balance may be maintained [17,18]. Thus, it was speculated that the reduction in Kt/V in the glucose group was related to the increased reuse of nitrogen (amino acid) due to the sufficient caloric supply. The reductions in glucose and amino acids during dialysis fail to explain the improvement of nutrition status in these patients.

Many hemodialysis patients were short of energy without serious amino acid deficiency, in agreement with the category of patients with energy malnourishment [8]. For these patients, the infusion of 250 mL of 50% glucose may be more effective than other complicated supplements, in agreement with our glucose group. Dietary supplement of 355 kcal per dialysis session improved the patients’ subjective global assessment scores and six minute walking test [19].

Some patients may benefit from the additional amino acid supplementation as our results confirmed that the amino acid group, which received both glucose and amino acids, had significantly higher albumin levels, in agreement with higher rates of albumin and protein synthesis reported by several studies [11,13,20,21]. Nutritional improvement that increased prealbumin levels to greater than 30 mg/L, regardless of treatment with oral supplementation or both oral supplementation and IDPN, reduced mortality and morbidity [22].

The limitations of this study are three-fold. The small sample size raises the possibility that these results may apply to a subpopulation of patients with CKD and undergoing hemodialysis. It should be noted that none of our patients were diabetic. Although we did not observe any withdrawal from the glucose treatment arm, 4% of patients in a different study had developed uncontrollable hyperglycemia [11]. Study results should be confirmed with a larger sample size in order to better understand any quality of life improvements in patients undergoing hemodialysis. Post-hoc power analyses have been criticized for interpreting negative study results, which implies either a small sample size or effect size, thus we will always get a low power for a negative study result. The result of SGA in our study had both: a small sample size (*N* = 10 or 12 in each group) and a small effect size (similar SGA distributions among groups). The observed power for detecting the difference of 70% and 40% between the glucose and control groups of SGA nutrition status of A was 0.294. The power could be obtained to more than 0.8 if the sample size increased to 40 in both groups. Secondly, this study investigated these three treatments on patients of Chinese origin at a single center and the results may vary depending on the ethnicity, common diet, oral supplementation, and food intake in the local cultures. Third, the length of the study was only nine months, and many patients require maintenance hemodialysis for multiple years. Thus, long term effects—both benefits and adverse events—may not be fully revealed by this study of nine months.

## 5. Conclusions

In conclusion, we found that a 250 mL infusion of 50% glucose solution replenished energy stores of non-diabetic patients undergoing hemodialysis for chronic kidney disease. This inexpensive and effective treatment may supply sufficient additional energy for numerous non-diabetic patients undergoing hemodialysis. It in turn may reduce their fatigue level, and improve their quality of life to some degree. Future studies are warranted to assess its long-term ability to replenish energy stores of non-diabetic hemodialysis patients.

## Figures and Tables

**Table 1:** Baseline characteristics of the three treatment groups.

		Control (*n* = 10)	Glucose (*n* = 10)	Amino Acid (*n* = 12)	*p*-Value
Age ^1^ (year)		71.80 (9.51)	74.00 (7.50)	69.83 (9.56)	0.560
Gender ^2^	Female	7 (70.0%)	4 (40.0%)	7 (58.3%)	0.458
	Male	3 (30.0%)	6 (60.0%)	5 (41.7%)	
Weight (kg)		62.2 (5.0)	57.3 (9.7)	60.5 (8.1)	0.379
BMI (kg/m^2^)		21.7 (1.9)	21.7 (3.2)	21.0 (2.5)	0.749
Dialysis duration (m)		21.3 ± 24.8	27.5 ± 20.1	24.7 ± 19.6	
Frequency of dialysis (times/week)		3	3	3	
Time of dialysis (h/week)		4	4	4	
Chronic glomerulonephritis		3	3	2	
Hypertensive nephropathy		5	5	7	
Obstructive nephropathy		1	2	3	
Polycystic kidney disease		1	0	0	

^1^ Data are presented by mean and standard deviation; ^2^ Data are presented by count with percentage.

**Table 2:** Comparison of changes in median of the SGA nutritional status for the three treatment groups.

		Control (*n* = 10)	Glucose (*n* = 10)	Amino Acid (*n* = 12)	*p*-Value
SGA baseline ^1^	A	6 (60.0%)	6 (60.0%)	7 (58.3%)	>0.999
	B	3 (30.0%)	3 (30.0%)	4 (33.3%)	
	C	1 (10.0%)	1 (10.0%)	1 (8.3%)	
SGA after treatment ^1^	A	4 (40.0%)	7 (70.0%)	8 (66.7%)	0.575
	B	5 (50.0%)	3 (30.0%)	3 (25.0%)	
	C	1 (10.0%)	0 (0.0%)	1 (8.3%)	
Energy intake ^2^ (kcal/kg)	Baseline	25.3 (19.5, 30.4)	23.9 (21.3, 27.7)	25.8 (20.9, 30.0)	0.871
	Post-treatment	24.9 (18.2, 28.5) ^‡^	28.2 (24.6, 31.0) ^‡^	25.1 (21.0, 29.1)	0.407
	Difference	−0.6 (−1.9, −0.4)	1.5 (0.8, 3.1) *	−0.7 (−1.9, 1.8) ^†^	0.024

^1^ Data are presented by count with percentage; ^2^ Data are presented by median and IQR (inter-quartile range, *i.e.*, the 25th and 75th percentiles). SGA, subjective global nutritional assessment: A—very mild risk to well-nourished; B—mild to moderate malnourished); C—severely malnourished. * *p* ≤ 0.05 compared to the control group; ^†^ *p* ≤ 0.05 compared to the glucose group; ^‡^ *p* ≤ 0.05 after treatment compared to baseline with groups.

**Table 3:** Comparison of median energy intake and biochemical indices for the three treatment groups.

		Control (*n* = 10)	Glucose (*n* = 10)	Amino Acid (*n* = 12)	*p*-Value
BUN (mmol/L)	Baseline	20.70 (15.40, 22.80)	19.40 (14.70, 21.20)	20.10 (15.55, 26.75)	0.550
	Post-treatment	18.30 (17.40, 21.40)	19.70 (18.10, 27.20) ^‡^	20.90 (16.85, 23.85)	0.650
	Difference	0.75 (−2.40, 3.5)	4.5 (−0.10, 6.10)	−1.95 (−4.15, 1.70)	0.081
nPCR (g/kg/day)	Baseline	0.87 (0.74, 0.99)	0.84 (0.72, 0.92)	0.84 (0.73, 1.11)	0.703
	Post-treatment	0.92 (0.86, 1.22)	0.90 (0.81, 1.13)	1.23 (1.0, 1.56) ^‡^	0.104
	Difference	0.15 (0.05, 0.36)	0.15 (0.02, 0.21)	0.31 (−0.07, 0.56)	0.629
Kt/V	Baseline	1.21 (1.15, 1.23)	1.22 (1.15, 1.39)	1.20 (1.09, 1.22)	0.402
	Post-treatment	1.28 (1.18, 1.35)	1.12 (0.97, 1.16) ^‡^	1.26 (1.12, 1.35) ^‡^	0.068
	Difference	0.12 (−0.04, 0.16)	−0.29 (−0.33, 0.01) *	0.14 (0.01, 0.21) ^†^	0.007
Hb (g/L)	Baseline	97.0 (93.0, 106.0)	100.0 (91.0, 107.0)	108.5 (90.5, 111.5)	0.706
	Post-treatment	106.5 (97.10, 126.0) ^‡^	110.0 (108.0, 140.0) ^‡^	121.0 (109.0, 128.5) ^‡^	0.365
	Difference	10.0 (3.0, 15.0)	13.5 (11.0, 27.0)	15.5 (8.5, 24.5)	0.422
TP (g/L)	Baseline	69.5 (65.60, 71.80)	68.55 (62.90, 75.10)	69.15 (65.90, 75.85)	0.640
	Post-treatment	69.30 (66.40, 71.70)	70.15 (66.40, 70.70)	70.40 (67.55, 76.25)	0.503
	Difference	0.60 (−1.60, 1.40)	0.5 (−1.0, 5.40)	0.15 (−2.30, 1.90)	0.915
Albumin (g/L)	Baseline	37.45 (35.5, 40.90)	35.35 (32.90, 38.40)	39.15 (36.95, 40.5)	0.109
	Post-treatment	37.10 (35.20, 38.10)	34.30 (33.40, 37.5)	38.70 (37.65, 41.15) ^†^	0.007
	Difference	−1.45 (−3.30, −0.30)	−2.25 (−3.60, 1.20)	−0.30 (−2.20, 1.20)	0.554
PA (mg/L)	Baseline	278.80 (229.90, 299.70)	210.40 (201.5, 261.30)	329.85 (234.95, 367.10)	0.073
	Post-treatment	267.10 (238.60, 283.90)	239.60 (228.40, 255.60)	340.5 (288.20, 348.40) *	0.034
	Difference	−13.30 (−17.80, 33.5)	32.5 (−0.30, 38.60)	9.25 (−11.80, 37.65)	0.427
TRF (g/L)	Baseline	1.80 (1.60, 2.10)	1.65 (1.5, 1.80)	1.75 (1.40, 2.25)	0.575
	Post-treatment	1.25 (1.10, 1.80) ^‡^	1.25 (0.80, 1.80) ^‡^	1.55 (0.85, 2.20) ^‡^	0.839
	Difference	−0.45 (−0.60, 0.0)	−0.45 (−0.5, 0.0)	−0.35 (−0.55, −0.15)	0.699
SCr (mcmol/L)	Baseline	928.0 (773.0, 992.0)	886.0 (754.0, 976.0)	939.5 (614.0, 1,100.5)	0.914
	Post-treatment	969.0 (805.0, 1,127.0)	1082.0 (1,041.0, 1,146.0)	1064.0 (719.5, 1,173.0) ^‡^	0.541
	Difference	56.5 (20.0, 165.0)	210.0 (97.0, 324.0)	73.0 (−4.0, 136.0)	0.266
TC (mmol/L)	Baseline	1.10 (0.80, 1.80)	1.30 (1.0, 1.60)	1.65 (1.20, 2.25)	0.530
	Post-treatment	1.45 (0.70, 1.90)	1.25 (0.90, 2.10)	1.70 (1.25, 2.10)	0.384
	Difference	0.25 (−0.10, 0.60)	0.10 (−0.30, 1.10)	0.30 (−0.55, 0.60)	0.996
TG (mmol/L)	Baseline	4.85 (3.60, 5.20)	4.70 (4.0, 5.70)	5.40 (3.70, 6.00)	0.742
	Post-treatment	4.10 (3.30, 4.80) ^‡^	4.45 (3.50, 4.90) ^‡^	4.30 (3.30, 6.15)	0.797
	Difference	−0.25 (−0.90, −0.10)	−0.30 (−0.60, −0.20)	−0.30 (−0.80, 0.45)	0.900
LDL-C (mmol/L)	Baseline	3.04 (2.10, 3.23)	2.96 (2.37, 4.26)	2.75 (2.09, 3.63)	0.915
	Post-treatment	2.13 (1.67, 2.82) ^‡^	2.71 (2.06, 3.06)	2.37 (1.86, 3.56)	0.326
	Difference	−0.49 (−0.84, −0.06)	−0.32 (−0.42, −0.05)	−0.15 (−0.54, 0.44)	0.316
hsCRP (mg/L)	Baseline	2.19 (1.27, 4.55)	6.13 (2.87, 12.76)	1.88 (1.04, 6.97)	0.146
	Post-treatment	2.47 (1.09, 5.06)	6.10 (3.29, 9.63)	3.30 (1.13, 8.60)	0.117
	Difference	−0.42 (−1.17, 1.28)	−0.98 (−4.51, 3.68)	0.30 (−2.62, 5.61)	0.528
Ca^2+^ (mmol/L)	Baseline	2.29 (2.25, 2.47)	2.22 (2.13, 2.26)	2.18 (2.15, 2.25)	0.050
	Post-treatment	2.54 (2.30, 2.72)	2.49 (2.34, 2.59) ^‡^	2.43 (2.35, 2.46) ^‡^	0.457
	Difference	0.20 (0.02, 0.34)	0.20 (0.07, 0.32)	0.22 (0.12, 0.29)	0.943
K^+^ (mmol/L)	Baseline	4.90 (4.10, 5.20)	4.20 (3.80, 4.90)	4.95 (4.35, 5.60)	0.150
	Post-treatment	4.75 (4.40, 5.10)	4.35 (3.50, 5.0)	4.50 (4.10, 5.10)	0.554
	Difference	−0.15 (−0.20, 0.20)	−0.20 (−0.50, 0.90)	−0.40 (−1.15, 0.30)	0.616
Ca × p (mg/dL)	Baseline	56.63 (35.13, 66.30)	59.13 (56.69, 63.50)	45.74 (34.53, 60.54)	0.569
	Post-treatment	59.07 (50.89, 70.82)	64.86 (45.46, 67.90)	72.18 (53.49, 92.36) ^‡^	0.767
	Difference	52.05 (14.0, 69.47)	33.09 (7.07, 57.49)	21.34 (6.68, 69.05)	0.682
TCO_2_ (mmol/L)	Baseline	21.50 (17.0, 23.0)	22.50 (21.0, 24.0)	18.0 (17.0, 20.50) ^†^	0.010
	Post-treatment	18.0 (16.0, 21.0) ^‡^	21.0 (20.10, 22.0)	18.0 (17.0, 22.50)	0.150
	Difference	−2.0 (−3.0, −1.0)	−1.0 (−3.0, 1.0)	0.50 (−1.50, 2.0)	0.108

Data are presented by median and IQR (inter-quartile range, *i.e.*, the 25th and 75th percentiles). * With a significant difference compared to the control group. ^†^ With a significant difference compared to the glucose group. ^‡^ With a significant change after treatment compared to baseline with groups. BUN, blood urea nitrogen; Ca^2+^, calcium ions; Ca × p, calcium-phosphorus product; Cr, creatinine; hsCRP, hypersensitive C-reactive protein; Hb, hemoglobin; K, potassium; Kt/V, indicates efficiency of dialysis; LDL-C, LDL-cholesterol; PA, Prealbumin; SCr, serum creatinine; TC, total cholesterol; TG, triglycerides; TCO_2_, total carbon dioxide, TP, total protein; TRF, transferrin.

**Table 4:** Comparison of median amino acid concentrations for the three treatment groups.

		Control (*n* = 10)	Glucose (*n* = 10)	Amino Acid (*n* = 12)	*p*-Value
Aspartic acid (ng/L)	Baseline	3.5 (3.0, 5.0)	3.5 (3.0, 4.0)	3.0 (3.0, 5.0)	0.964
	Post-treatment	3.0 (2.0, 3.0)	3.0 (2.0, 3.0)	2.0 (2.0, 2.5) ^‡^	0.236
	Difference	−1.0 (−2.0, 0.0)	−0.5 (−2.0, 0.0)	−1.0 (−2.0, −1.0)	0.548
Glutamic acid (ng/L)	Baseline	93.0 (77.0, 111.0)	67.5 (50.0, 94.0)	75.0 (59.0, 115.0)	0.209
	Post-treatment	78.0 (71.0, 94.0)	82.5 (68.0, 98.0)	69.5 (64.5, 85.5)	0.536
	Difference	−3.5 (−31.0, 6.0)	25.5 (−4.0, 34.0)	−9.5 (−49.0, 10.0)	0.089
Asparagine (ng/L)	Baseline	43.5 (37.0, 56.0)	34.5 (31.0, 50.0)	43.0 (35.0, 76.5)	0.432
	Post-treatment	46.0 (31.0, 67.0)	53.5 (47.0, 64.0) ^‡^	36.0 (26.5, 45.5) ^†^	0.023
	Difference	3.5 (−10.0, 21.0)	15.0 (7.0, 27.0)	−13.0 (−32.0, 10.0) ^†^	0.006
Serine (ng/L)	Baseline	105.5 (84.0, 113.0)	84.0 (75.0, 110.0)	101.5 (66.5, 154.5)	0.551
	Post-treatment	103.0 (78.0, 134.0)	110.5 (85.0, 122.0)	93.5 (73.5, 111.5)	0.362
	Difference	4.0 (−31.0, 17.0)	17.5 (−3.0, 43.0)	−17.0 (−54.5, 21.5)	0.222
Glutamine (ng/L)	Baseline	527.0 (442.0, 655.0)	453.0 (431.0, 525.0)	590.5 (431.5, 673.0)	0.312
	Post-treatment	504.5 (344.0, 609.0)	570.0 (485.0, 606.0) ^‡^	522.0 (389.0, 640.5)	0.614
	Difference	−62.0 (−132.0, −16.0)	62.5 (12.0, 158.0) *	−51.5 (−258.5, 66.0) ^†^	0.028
Glycine (ng/L)	Baseline	319.5 (256.0, 414.0)	245.0 (210.0, 344.0)	300.5 (242.0, 398.5)	0.220
	Post-treatment	304.0 (206.0, 347.0)	349.5 (269.0, 409.0) ^‡^	283.0 (238.0, 332.0)	0.169
	Difference	−37.0 (−97.0, 5.0)	59.5 (2.0, 113.0) *	−32.0 (−135.5, 42.5) ^†^	0.011
Histidine (ng/L)	Baseline	68.5 (53.0, 90.0)	59.5 (48.0, 68.0)	84.0 (60.5, 101.0)	0.071
	Post-treatment	80.0 (66.0, 88.0)	77.0 (58.0, 79.0)	65.0 (46.5, 77.5)	0.362
	Difference	12.5 (−21.0, 22.0)	14.0 (0.0, 15.0)	−14.0 (−40.5, 6.0) *^,†^	0.027
Threonine (ng/L)	Baseline	125.0 (120.0, 162.0)	102.5 (92.0, 126.0)	111.0 (94.5, 178.0)	0.242
	Post-treatment	123.0 (98.0, 167.0)	132.0 (107.0, 156.0)	106.5 (68.0, 119.5)	0.095
	Difference	−9.5 (−37.0, 18.0)	27.5 (11.0, 50.0)	−18.0 (−75.5, 12.5) ^†^	0.045
Citrulline (ng/L)	Baseline	113.0 (84.0, 124.0)	81.0 (71.0, 115.0)	89.0 (59.0, 137.0)	0.480
	Post-treatment	110.5 (97.0, 118.0)	112.0 (102.0, 117.0)	79.5 (64.5, 96.0)	0.081
	Difference	−5.0 (−11.0, 13.0)	15.5 (−12.0, 59.0)	−12.5 (−47.5, 15.5)	0.173
Alanine (ng/L)	Baseline	420.0 (331.0, 540.0)	326.5 (261.0, 384.0)	365.5 (260.5, 642.5)	0.387
	Post-treatment	368.5 (281.0, 398.0)	390.5 (346.0, 464.0) ^‡^	322.0 (224.5, 403.5) ^‡^	0.292
	Difference	−67.5 (−97.0, 23.0)	31.0 (6.0, 135.0)	−53.0 (−156.0, −31.0) ^†^	0.009
Taurine (ng/L)	Baseline	91.5 (43.0, 109.0)	69.5 (30.0, 119.0)	54.0 (41.0, 110.0)	0.897
	Post-treatment	92.5 (77.0, 122.0)	111.5 (83.0, 122.0)	106.5 (84.5, 162.0)	0.752
	Difference	20.5 (−23.0, 61.0)	41.5 (−29.0, 53.0)	50.5 (−26.0, 74.0)	0.914
Arginine (ng/L)	Baseline	61.0 (50.0, 86.0)	47.0 (38.0, 72.0)	51.5 (42.0, 106.0)	0.404
	Post-treatment	49.5 (21.0, 70.0) ^‡^	47.0 (42.0, 118.0)	43.5 (27.0, 69.0)	0.568
	Difference	−24.5 (−32.0, 5.0)	4.0 (−27.0, 54.0)	−21.5 (−63.0, 5.0)	0.082
Tyrosine (ng/L)	Baseline	39.0 (34.0, 40.0)	36.0 (33.0, 50.0)	42.5 (30.5, 71.0)	0.570
	Post-treatment	38.0 (30.0, 44.0)	43.0 (39.0, 54.0)	32.0 (26.0, 41.0)	0.141
	Difference	2.0 (−12.0, 15.0)	9.5 (−1.0, 12.0)	−10.5 (−28.5, 5.0)	0.080
Valine (ng/L)	Baseline	208.5 (183.0, 247.0)	178.0 (166.0, 228.0)	217.0 (199.5, 315.5)	0.325
	Post-treatment	206.0 (174.0, 239.0)	190.0 (172.0, 268.0)	185.0 (147.5, 256.0)	0.801
	Difference	4.0 (−44.0, 50.0)	39.5 (−6.0, 52.0)	−36.0 (−120.5, 31.5)	0.255
Methionine (ng/L)	Baseline	15.5 (12.0, 20.0)	13.0 (11.0, 15.0)	19.0 (12.5, 27.0)	0.086
	Post-treatment	12.0 (11.0, 19.0)	15.0 (12.0, 26.0)	11.5 (9.0, 17.0) ^‡^	0.268
	Difference	−2.0 (−4.0, 2.0)	4.5 (−1.0, 12.0)	−5.5 (−12.0, 0.0) ^†^	0.009
Trytophan (ng/L)	Baseline	22.5 (19.0, 30.0)	22.0 (16.0, 35.0)	22.0 (18.0, 37.5)	0.956
	Post-treatment	20.5 (17.0, 29.0)	28.0 (24.0, 35.0)	15.0 (13.5, 24.5) ^†^	0.039
	Difference	−1.0 (−9.0, 3.0)	6.5 (−1.0, 10.0)	−6.0 (−14.0, 1.0)	0.055
Phenylalanine (ng/L)	Baseline	94.5 (65.0, 109.0)	75.0 (59.0, 93.0)	81.5 (58.0, 108.5)	0.364
	Post-treatment	87.5 (64.0, 110.0)	89.5 (75.0, 102.0)	64.5 (47.0, 79.0) *^,†,‡^	0.022
	Difference	−1.0 (−13.0, 9.0)	21.0 (−15.0, 35.0)	−20.5 (−52.0, −1.5) ^†^	0.020
Isoleucine (ng/L)	Baseline	71.0 (45.0, 92.0)	49.0 (48.0, 54.0)	63.5 (52.0, 113.0)	0.145
	Post-treatment	62.5 (36.0, 76.0)	54.5 (42.0, 81.0)	51.0 (37.5, 70.5)	0.836
	Difference	−3.0 (−19.0, 8.0)	18.5 (−10.0, 31.0)	−10.0 (−34.5, 12.5)	0.108
Ornithine (ng/L)	Baseline	48.0 (40.0, 64.0)	55.0 (36.0, 87.0)	65.0 (37.5, 80.0)	0.780
	Post-treatment	62.0 (46.0, 96.0)	61.5 (55.0, 77.0)	45.0 (36.0, 65.5)	0.118
	Difference	8.5 (2.0, 30.0)	7.0 (−11.0, 36.0)	−16.5 (−43.0, 20.0)	0.243
Leucine (ng/L)	Baseline	92.0 (83.0, 147.0)	88.0 (78.0, 115.0)	114.5 (90.5, 183.0)	0.182
	Post-treatment	109.5 (82.0, 127.0)	89.5 (75.0, 147.0)	91.0 (75.0, 122.5)	0.844
	Difference	12.5 (−20.0, 22.0)	24.5 (−1.0, 44.0)	−28.0 (−57.5, 28.0)	0.142
Lysine (ng/L)	Baseline	118.5 (71.0, 143.0)	99.0 (59.0, 113.0)	86.0 (65.0, 145.5)	0.532
	Post-treatment	107.5 (78.0, 121.0)	105.5 (86.0, 129.0) ^‡^	86.0 (49.0, 140.5)	0.366
	Difference	−7.0 (−45.0, 46.0)	32.0 (11.0, 39.0)	−9.0 (−87.0, 36.5)	0.311
Proline (ng/L)	Baseline	344.0 (303.0, 392.0)	238.5 (205.0, 276.0) *	302.0 (259.5, 348.0) ^†^	0.014
	Post-treatment	278.5 (191.0, 322.0)	219.0 (110.0, 274.0)	182.0 (161.0, 311.0) ^‡^	0.412
	Difference	−73.5 (−181.0, 27.0)	−13.0 (−100.0, 67.0)	−89.0 (−155.5, −13.0)	0.453

* *p* ≤ 0.05 compared to the control group; ^†^ *p* ≤ 0.05 compared to the glucose group; ^‡^ *p* ≤ 0.05 after treatment compared to baseline within groups.

## Abbreviations

The following abbreviations are used in this manuscript:

BMI	body mass index
BUN	blood urea nitrogen
Ca^2+^	calcium ions
Ca × p	Calcium × phosphate levels
Cr	creatinine
Hb	hemoglobin
hsCRP	hypersensitive C-reactive protein
IDPN	intradialytic parenteral nutrition
IQR	interquartile range
K^+^	potassium ions
Kt/V	indicates efficiency of dialysis
LDL-C	low density lipoprotein-cholesterol
PA	prealbumin
PEW	protein-energy wasting
SCr	serum creatinine
SGA	subjective global nutritional assessment
TC	total cholesterol
TCO_2_	total carbon dioxide
TG	triglyceride
TP	total protein
TRF	transferrin
